# Use of Bioimpedance to Assess Changes in Hemodynamics During Acute Administration of CPAP

**DOI:** 10.4021/cr18w

**Published:** 2011-03-25

**Authors:** Genevieve C. Digby, Helen S. Driver, Michael Fitzpatrick, Glorianne Ropchan, Christopher M. Parker

**Affiliations:** a Department of Medicine, Queen’s University, Kingston, Ontario, Canada; b Department of Surgery, Queen’s University, Kingston, Ontario, Canada; c Department of Physiology, Queen’s University, Kingston, Ontario, Canada; d Sleep Disorders Laboratory, Kingston General Hospital, Kingston, Ontario, Canada

**Keywords:** Continuous positive airway pressure, Obstructive sleep apnea, Transthoracic electrical bioimpedance, Pulmonary artery catheters

## Abstract

**Background:**

Attempts to investigate the mechanisms by which continuous positive airway pressure (CPAP) therapy improves heart function in patients with obstructive sleep apnea (OSA) have been limited by the lack of non-invasive methods to assess cardiac performance. We used transthoracic electrical bioimpedance (TEB) to assess acute hemodynamic changes including heart rate (HR), stroke volume (SV), cardiac output (CO) and cardiac index (CI) during PAP titration in (1) post-operative cardiac surgery patients, (2) patients with severe OSA, and (3) normal healthy volunteers.

**Methods:**

Post-operative cardiac surgery patients were studied via TEB and pulmonary artery catheter (PAC) during acute titration of positive end-expiratory pressure (PEEP) while mechanically ventilated. Patients with severe OSA were studied non-invasively by TEB during acute CPAP titration in supine stage 2 sleep, and normal subjects while awake and recumbent.

**Results:**

In post-operative cardiac surgery patients (n = 3), increasing PEEP to 18 cmH_2_O significantly reduced SV and CI relative to baseline. There was no difference between TEB and PAC in terms of ability to assess variations in hemodynamic parameters. In patients with severe OSA (n = 3), CPAP titration to optimal pressure to alleviate obstructive apneas reduced HR, SV, CO and CI significantly compared to without CPAP. In three healthy subjects, maximal tolerated CPAP reduced SV and CO significantly compared to baseline.

**Conclusions:**

Acute administration of CPAP causes a decrease in CO and CI, apparently a consequence of a reduction in SV. TEB appears to be an accurate and reproducible non-invasive method of detecting changes in hemodynamics.

## Introduction

Obstructive sleep apnea (OSA) is a common sleep-related breathing disorder associated with an increased risk of cardiovascular disease [1]. OSA is characterized by repetitive occlusions of the upper airway during sleep, the physiological consequences of which include arterial hypoxemia and hypercapnia, endothelial dysfunction, and sympathetic activation, which in turn lead to an increased risk of cardiovascular morbidity and mortality [2]. Continuous positive airway pressure (CPAP) therapy is the mainstay of treatment of moderate-to-severe OSA, and may significantly decrease the risk of cardiovascular disease [3, 4]. In fact, studies involving OSA patients with congestive heart failure (CHF) have shown that chronic use of nocturnal CPAP therapy improves left ventricular systolic function [5-7], decreases sympathetic activity [5], reduces systolic blood pressure [6] and improves quality of life [5]. CPAP therapy studies involving OSA patients without CHF have also shown significant improvements in ventricular structural and functional changes including decreased ventricular wall thickness, decreased right ventricular dilation and improved contractility [8, 9]. It has been hypothesized that most of the benefit of CPAP therapy is due to the prevention of both the large negative swings in intrathoracic pressure (which increase left ventricular afterload) and the associated arterial oxygen desaturation [10].

Attempts to further investigate the mechanisms by which CPAP therapy improves heart function have been limited by the availability of methods to assess cardiac performance. Pulmonary artery catheterization (PAC) has traditionally been used to measure hemodynamic variables, and is also considered to be the clinical gold standard for cardiac output monitoring [11]. However, the invasive nature of this device and its associated risks render it a less than ideal candidate for routine investigation of the OSA patient population. As far as non-invasive measures are concerned, echocardiography is one method that has gained popularity for this purpose. Studies using echocardiography have shown that long-term use of CPAP by OSA patients reduces resting heart rate (HR) and increases stroke volume (SV) [6, 8]. Yet, the extent to which cardiac output (CO), or its sub-components (HR and SV), can be acutely influenced by CPAP therapy remains unclear, as do the effects on these parameters incurred by apneic events during various sleep stages.

Recently, the use of transthoracic electrical bioimpedance (TEB) devices has gained popularity in the assessment of hemodynamics in various clinical and research settings. The accuracy and reproducibility of the results have repeatedly been confirmed in studies demonstrating that CO measured by TEB correlate closely, and with clinically acceptable agreement and precision, compared with CO measurements obtained by pulmonary artery thermodilution among both stable and unstable post-cardiac surgery patients [12, 13]. Advantages of TEB are that it is non-invasive in nature, it is not as time consuming as echocardiography, and does not require the training or skill required for either PAC or echocardiography [14]. In light of these advantages, TEB has become an increasingly popular method of assessing the hemodynamic status of patients in several different clinical scenarios, especially that of heart failure [15-18].

Few studies, however, have investigated the use of TEB in patients with OSA. Stoohs et al [19] used non-invasive electrical bioimpedance to investigate the hemodynamic changes that occur during OSA. Specifically, they focused on the hemodynamic changes that occur during individual apneic events in REM and NREM sleep. In spite of this study, further investigation regarding the hemodynamic changes that occur during OSA by using TEB has been limited, and none have used TEB to investigate the effect of CPAP therapy on hemodynamics.

We used a bioimpedance device that has previously been validated as a method of monitoring CO as compared to the direct Fick method and to thermodilution [20, 21], to non-invasively measure hemodynamic variables in three populations undergoing titration of positive airway pressure: (i) normal subjects, (ii) patients with severe OSA, and (iii) post-operative cardiac surgery patients. In this latter group, hemodynamic measurements were compared to those obtained invasively using a PAC in order to offer a comparison of the TEB device versus the currently accepted gold standard (PAC).

## Materials and Methods

This study was approved by the Research Ethics Board at Queen’s University. All participating subjects provided informed consent. Normal subjects were recruited using word-of-mouth, whereas patients suspected of having severe OSA and those undergoing elective cardiac surgery were recruited from Sleep Clinics and from Cardiac Surgery Clinics, respectively.

### Bioimpedance device

Non-invasive measurements of hemodynamic parameters (including HR, SV, CO and CI) were obtained using TEB (PhysioFlow™; Manatec Biomedical, Paris, France). This device utilizes bioimpedance to derive indices of CO and SV by placing six adhesive electrodes on the patients’ thorax and neck (two electrodes on the neck, two electrodes on the spine, one electrode in the mid-axillary line and one over the sternum). The device was set so that acquisition of data was averaged over 5 heart cycles. Additional output included HR and cardiac index (CI). The CI was calculated by dividing the measured CO by the patients’ body surface area (BSA), where BSA was calculated using the Dubois-Dubois formula: BSA (m2) = 0.007184 × weight (kg)0.425 × height (cm)0.725.

#### The effect of CPAP on hemodynamics in post-operative CABG patients

Patients undergoing elective cardiac surgery with the anticipated placement of a PAC were eligible to participate. Post-operatively, patients were managed according to current routine practice. Once hemodynamic stability was obtained, baseline hemodynamic parameters (HR, SV, CO, CI) were determined both invasively (using the thermodilution technique via the PAC) and non-invasively (using TEB) while the patient was mechanically ventilated (tidal volume 10 cc/kg) with a positive end-expiratory pressure (PEEP) of 5 cmH_2_O. PEEP was then sequentially increased to 10, 15, and 18 cmH_2_O and was finally returned to a baseline of 5 cmH_2_O. After each change in PEEP, hemodynamic variables were measured both invasively and non-invasively. Thermodilution assessment of cardiac parameters was performed as per accepted techniques; a mean of three measurements at each level of PEEP was reported. Data acquired by the bioimpedance device was exported to a Microsoft Excel Spreadsheet for determination of hemodynamic variables by averaging values obtained in the last 120 seconds prior to changing PEEP; each individual measurement generated by TEB represents an average of 5 heart cycles. For hemodynamic variables, the percentage change was calculated using the following formula: measure obtained at a given PEEP, minus baseline value (PEEP = 5 cm H_2_O), divided by baseline value.

#### The effect of CPAP on hemodynamics in severe OSA patients during Stage 2 sleep

Patients suspected of having severe OSA and scheduled for a split-night (diagnostic to CPAP therapy titration) sleep study were screened for entry. Patients found to meet the criteria for severe OSA (defined as an apnea-hypopnea index of > 30/h) [22] during the diagnostic segment of the sleep study were eligible to participate. The routine overnight sleep study (polysomnography) included 4 electroencephalogram channels (C4-A1, C3-A2, O2-A1, O1-A2), 2 electrooculogram channels (ROC-A1, LOC-A2), chin electromyogram, intercostal electromyogram, electrocardiogram, chest and abdominal movement (piezo belts), finger pulse oximetry, anterior tibialis electromyogram, and vibration snore sensor; airflow was measured using both a thermocouple and a nasal cannula pressure transducer. A video camera mounted in the wall of the bedroom was used to monitor the subject and record body position. Polysomnographic recordings were made on Sandman SD32 (Tyco Healthcare) digital systems concurrently with bioimpedence recording. The same experienced scorer scored all the sleep records in 30-second epochs according to standardized criteria [23]. Obstructive apneas and hypopneas were scored using the criteria from the American Academy of Sleep Medicine Task Force [24]. Events were scored as apneas when a > 50% decrease in airflow was recorded, or hypopneas when a clear reduction in amplitude of the airflow signal (compared to stable breathing during the 2 minutes preceding the event), occurred associated with an arousal and/or a greater than 3% reduction in oxygen saturation (SaO_2_), and the event lasted for at least 10 seconds. Arousals were scored based on American Sleep Disorders Association criteria [22]. Arousals had to be preceded by at least 10 seconds of sleep, have an electroencephalogram frequency shift to alpha or theta for at least 3 seconds and up to 15 seconds, and be associated with concurrent increased electromyogram tone in REM sleep. An apnea-hypopnea index (AHI) and the respiratory disturbance (RDI), which included apneas, hypopneas, and snore arousals for the number of events per hour of sleep, were calculated for the diagnostic study and during CPAP titration.

Data acquired by the bioimpedance device was exported to a Microsoft Excel Spreadsheet. Obstructive events (hypopneas and apneas with and without arousals) were identified using the scored sleep studies. Only those events occurring during Stage 2 (NREM) sleep and in the supine body position were included in the analysis in order to limit confounding variables. Matched time-periods corresponding to each obstructive event was located in the bioimpedance data. The values for HR, SV, and CO, as obtained every 5 heart cycles from the bioimpedance device, during each obstructive period were averaged. Each obstructive event was matched with a corresponding period of equivalent duration, also occurring in Stage 2 (NREM) sleep and in the supine body position, when there were no obstructive events or flow limitations, both prior to CPAP administration and at an optimal pressure where obstructive events were absent. Matched periods were chosen on the basis that there was at least 10 seconds separating the last obstructive period from the selected time-matched obstruction-free period. Average HR, SV and CO during these time-matched periods were calculated.

#### The effect of CPAP on hemodynamics in normal, healthy subjects

Healthy individuals with no history of cardiac or respiratory disease, no OSA, and no significant chronic medical conditions were recruited. Subjects were studied while awake, during the daytime, to evaluate the acute effects of CPAP therapy. They were fitted with a full-face CPAP mask and were studied during recumbency with the head of the bed raised to 25 degrees. The bioimpedance electrodes were attached and the recording was started initially with no CPAP pressure (i.e. 0 cmH_2_O) as the baseline value. After 3 - 5 minutes of lying still, and after steady state of hemodynamic parameters was attained, CPAP was initiated at a pressure of 4 cmH_2_O. In 2 - 3 minute intervals, the CPAP pressure was increased in 2 cmH_2_O increments to the maximal tolerated pressure of each subject (14 cmH_2_O, 16 cmH_2_O and 18 cmH_2_O, respectively). The pressure was then decreased back to 4 cmH_2_O, and in the subsequent 2 - 3 minute interval, CPAP was terminated.

TEB data were exported to an Excel spreadsheet and hemodynamic indices were averaged over 2 minutes of baseline when the patient was wearing the CPAP mask but with no applied pressure. After each change in CPAP pressure, the last 1.5 minutes of the interval leading up to the subsequent change in CPAP pressure was used to determine the average of HR, SV and CO for that interval. Once CPAP was discontinued after the final interval, another 2 minutes of data was acquired after 30 seconds had passed following the discontinuation of CPAP. The percentage change in hemodynamic parameters was calculated using the following formula: measurement obtained at a given CPAP pressure, minus baseline value, divided by baseline value.

### Statistical analysis

Data are expressed as mean ± SD. Student’s T-test was used to determine significance. For normal subjects and those with severe OSA, P < 0.05 was considered to be significant. For post-operative cardiac surgery patients, a Bonferonni correction factor was applied in order to correct for multiple comparisons and thus P < 0.0125 was considered significant. For all analyses, P < 0.1 was considered to represent a trend.

## Results

### The effect of CPAP on hemodynamics in post-operative CABG patients

**Figure 1:**
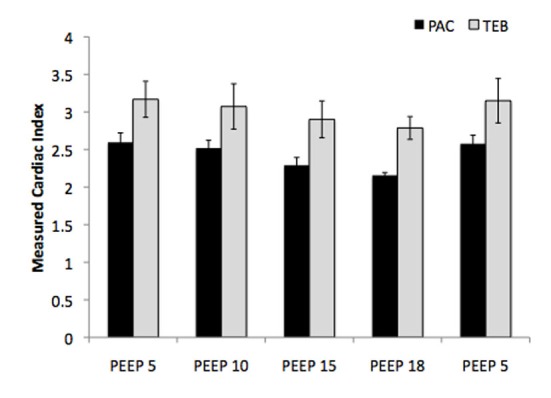
Numerical measurements of cardiac index with increasing PEEP as measured by PAC and TEB in post-operative cardiac surgery patients. PAC, pulmonary artery catheter; PEEP, positive end-expiratory pressure; TEB, transthoracic electrical bioimpedance.

Patients (n = 3) undergoing cardiac surgery (coronary artery bypass grafting) had a mean age of 64 (range 57 - 74) with either preserved (grade I) or mildly impaired (grade II) left-ventricular function as assessed intraoperatively with transesophageal echocardiography. Figure 1 presents the numerical values of CI as measured by PAC and TEB in the setting of increasing PEEP in post-operative cardiac surgery patients. The values measured by TEB were slightly, and consistently, higher than those measured by PAC. Though the absolute numbers may differ, the observed trend of decreasing CI with increasing PEEP is reproducible with both devices, and the percentage change in hemodynamic values as measured by each device was found to be minimally different.

**Figure 2:**
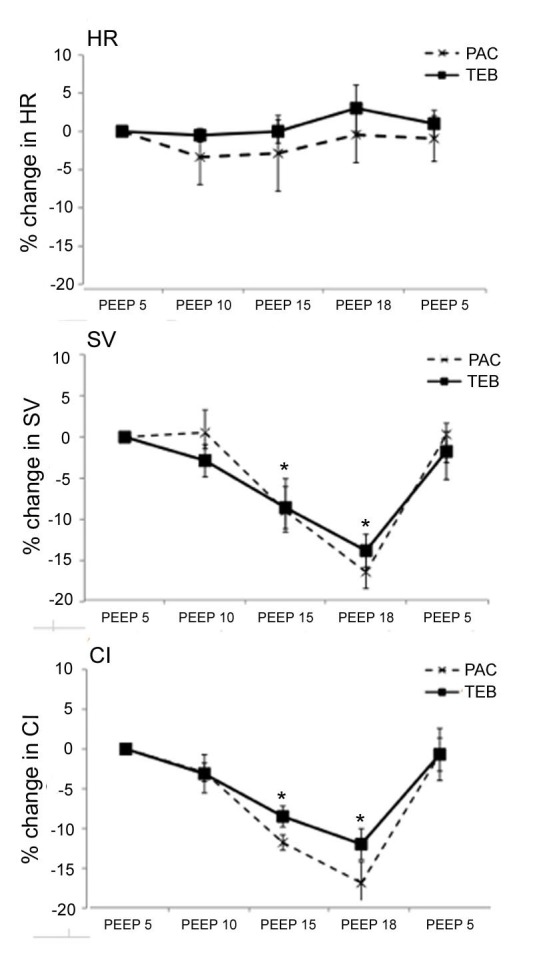
Percentage change in hemodynamic parameters with increasing PEEP in post-operative CABG patients. PAC, pulmonary artery catheter; PEEP, positive end-expiratory pressure; TEB, transthoracic electrical bioimpedance.

Figure 2 presents the percentage change in HR, SV and CI with increasing levels of PEEP (from 5 cmH_2_O to 18 cmH_2_O) as measured by both PAC and TEB. Compared to baseline (PEEP 5 cmH_2_O), there was a significant reduction in SV and CI with increasing PEEP returning to baseline levels when the PEEP was subsequently decreased back to the baseline (5 cmH_2_O). HR remained unaltered by changing PEEP.

### The effect of CPAP on hemodynamics in severe OSA patients during Stage 2 sleep

Three patients with severe OSA (2M, 1F) aged mean 53.3 ± 20.8 years with BMI of 36.3 ± 7.6 kg/m^2^ were studied. The participants had a mean respiratory disturbance index of 57.9 ± 39.4/hr. Data from 88 obstructive hypopneas of an average of 23.3 ± 7.6 seconds in duration (total of 2048 seconds) were compiled from three patients in the supine position with a CPAP pressure of 0 cmH_2_O. Data from 30 individual time periods of an average of 28.4 ± 7.4 seconds in duration (total of 853 seconds) were compiled from two of these same patients in the supine position at the same CPAP pressure when no apneas occurred. One patient was excluded because there were no periods of sufficient duration during which no apneas occurred when CPAP pressure was 0 cmH_2_O (i.e., obstructive events were found to occur almost continuously prior to CPAP therapy). Fifty-eight individual time periods of an average of 23.3 ± 6.7 seconds in duration (total of 1353 seconds) were compiled from all three patients once CPAP had been titrated to an optimal pressure at which apneas were eliminated in the supine position (corresponding to a pressure of 5.0 cmH_2_O, 8.0 cmH_2_O, and 9 cmH_2_O respectively).

The results are presented in Table 1. In patients with severe OSA, CPAP titration to optimal pressure to alleviate obstructive apneas (median 8 cmH_2_O, range 5 - 9 cmH_2_O) reduced HR significantly from 71.8 ± 7.1 min^-1^ to 60.9 ± 5.2 min^-1^ (P < 0.0001); SV from 93.5 ± 4.8 mL to 83.1 ± 6.9 mL (P < 0.0001), CO from 6.7 ± 0.4 L/min to 5.1 ± 0.6 L/min (P < 0.0001) and CI from 2.9 ± 0.1 L/min/m^2^ to 2.3 ± 0.3 L/min/m^2^ (P < 0.0001) compared to without CPAP but in the absence of apneas.

**Table 1:** Effect of CPAP on Hemodynamics in Three Patients With Severe OSA

	No CPAP, Apneas (n = 68)	No CPAP, No Apneas (n = 30)	Max CPAP, No Apneas (n = 58)	P value (Max CPAP, No Apneas vs. No CPAP, No Apneas)
HR	71.9 ± 7.1	71.8 ± 2.7	60.9 ± 5.2	< 0.0001
SV	90.2 ± 12.6	93.5 ± 4.8	83.1 ± 6.9	< 0.0001
CO	6.5 ± 1.1	6.7 ± 0.4	5.1 ± 0.6	< 0.0001
CI	2.8 ± 0.4	2.9 ± 0.1	2.3 ± 0.3	< 0.0001

Values are mean ± SD. All values represent NREM (stage 2) sleep in the supine position. CI, cardiac index; CO, cardiac output; HR, heart rate; SV, stroke volume; n, total number of individual time periods analyzed from all three patients.

### The effect of CPAP on hemodynamics in normal, healthy subjects

**Figure 3:**
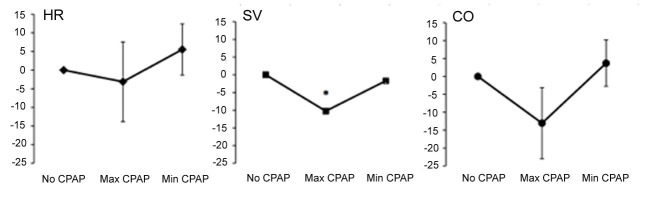
Percentage change in hemodynamic parameters with CPAP administration. Max CPAP, maximal tolerated CPAP (12 cmH_2_O, 14 cmH_2_O, and 18 cmH_2_O in each respective patient); Min CPAP, minimal CPAP pressure reached before end of study (0 cmH_2_O, 0 cmH_2_O and 4 cmH_2_O for each respective patient).

Data for normal, healthy subjects (1M, 2F aged mean 31.0 ± 4.4 years with BMI of 23.1 ± 3.0 kg/m^2^) are presented in Figure 3. The percentage change in HR, SV and CO from baseline prior to CPAP therapy (0 cmH_2_O), at the maximal tolerated CPAP pressure (14 cmH_2_O, 16 cmH_2_O and 18 cmH_2_O, respectively) and at the minimal CPAP pressure achieved prior to termination of bioimpedance recordings (0 cmH_2_O, 0 cmH_2_O and 4 cmH_2_O, respectively) are shown.

Compared to baseline, while HR remained unchanged, there was a significant reduction in SV (by 10.3 ± 0.4%, P < 0.0001) and a trend towards a reduction in CO (by 13.0 ± 9.9%, P = 0.085) at maximal CPAP pressures. On decreasing CPAP from the maximal tolerated pressure to the minimal pressure achieved prior to termination of bioimpedance recordings, both SV and CO returned to baseline values.

## Discussion

This study used a non-invasive bioimpedance device to assess the acute hemodynamic changes during positive airway pressure titration in post-operative cardiac surgery patients with PACs, patients with severe OSA, and normal healthy subjects. To our knowledge, this study represents the first reported use of electrical bioimpedance in assessing hemodynamic variables in patients undergoing positive airway pressure titration.

We found that the administration of acute positive airway pressure consistently leads to a decrease in CO and SV across the three groups investigated. The effect was also found to be directly proportional to the amount of CPAP delivered. These findings are similar to those reported using echocardiography. For example, a recent study by Johnson et al [25] using echocardiography similarly showed that acute CPAP administration decreased SV and left ventricular ejection fraction in stable CHF patients with OSA, and Liston et al [26] also observed a decrease in both CO and SV with acute CPAP therapy in stable CHF patients. In contrast, however, other studies have reported either an increase or no change in SV and CO with acute CPAP therapy [27, 28].

It has been suggested that acute CPAP therapy increases intrathoracic pressure and may therefore decrease systemic venous return. At higher levels of positive airway pressure, it has also been postulated that pulmonary vascular resistance may increase, which in turn would increase right ventricular afterload; at extremes this may translate into reduced right ventricular performance with subsequent reduction in left ventricular preload and SV [29-31]. In light of these findings and those of the current study, the observed tendency for CO to decrease with acute CPAP appears to be a result of the inherent physiological effects of CPAP on the cardio-respiratory system and does not appear limited to a specific disease process or patient population.

Interestingly, in our study, the observed reduction in CO seemed to be largely a consequence of a reduction in SV, which was observed across all patient populations. In patients with severe OSA, administration of CPAP titrated to optimal pressure to eliminate obstructive apneas was associated with a reduction in HR as compared to stage- and position-matched control epochs without CPAP. Similar to our results, acute administration of CPAP therapy during sleep to patients with concurrent moderate-to-severe OSA and congestive heart failure resulted in a reduction in HR with CPAP [25]. Furthermore, administration of CPAP therapy in patients with severe OSA has previously been shown to attenuate activation of the sympathetic nervous system [32]; we speculate that this may have contributed to the reduction in HR observed in our OSA patient population with the administration of CPAP. In contrast, in post-operative cardiac surgery patients, no change in HR was observed during titration of PEEP; however it must be recognized that these patients were receiving temporary cardiac pacing (at a rate of 80 min^-1^) as part of routine post-operative care, so this may have obscured the ability to detect changes in HR with PEEP titration. Importantly, however, since the heart rate did not change (since the patients were paced) during application of positive end-expiratory pressure, any observed reduction in cardiac output with PEEP must have resulted from a reduction in stroke volume.

By investigating the hemodynamic changes that occur with positive pressure titration in post-operative CABG patients using both TEB and PAC, it was possible to examine agreement and reproducibility of these two devices. Pulmonary artery catheterization is generally considered to be the clinical gold standard for CO monitoring, but this invasive method is not without complications and, thus, its use has declined over the past several years [11]. This study found that TEB consistently over-estimated CI in comparison to PAC. However, though the measured absolute values differed, the devices were remarkably similar in their measurements of percentage change in hemodynamic parameters across all levels of CPAP titration. There was minimal difference between TEB and PAC in terms of ability to assess these variations in hemodynamic parameters when expressed as percentage change. In light of the invasive limitations of PACs, TEB may be considered a useful and accurate alternative for assessing acute changes in hemodynamic parameters. However, an obvious limitation of this study is the small sample size in each of the three groups.

These findings offer a better understanding of the acute effects of CPAP therapy on the cardiovascular system, yet the mechanisms by which CPAP therapy improves cardiovascular outcomes are still unclear. Further studies are warranted to better define the impact of CPAP therapy on hemodynamic parameters, both acutely and chronically. Meanwhile, our study suggests that TEB may be equally efficacious in assessing acute changes in hemodynamic parameters as PACs, and the non-invasive nature of this device renders it a very attractive alternative to conduct further studies in this field.
